# *In situ* MEMS testing: correlation of high-resolution X-ray diffraction with mechanical experiments and finite element analysis

**DOI:** 10.1080/14686996.2017.1282800

**Published:** 2017-03-31

**Authors:** Andreas Schifferle, Alex Dommann, Antonia Neels

**Affiliations:** ^a^ Helbling Technik AG, Aarau, Switzerland; ^b^ Empa, Swiss Federal Laboratories for Materials Science and Technology, Center for X-ray Analytics, Dübendorf, Switzerland

**Keywords:** High-resolution X-ray diffraction (HRXRD), reciprocal space mapping, *in situ* material characterization, single crystal silicon, finite element analysis (FEA), 10 Engineering and Structural materials, 106 Metallic materials, 201 Electronics / Semiconductor / TCOs, 208 Sensors and actuators, 303 Mechanical / Physical processing, 401 1st principle calculations, 504 X-ray / Neutron diffraction and scattering, 600 Advance material characterization, 600 Mechanical testing and Finite Element Analysis

## Abstract

New methods are needed in microsystems technology for evaluating microelectromechanical systems (MEMS) because of their reduced size. The assessment and characterization of mechanical and structural relations of MEMS are essential to assure the long-term functioning of devices, and have a significant impact on design and fabrication.

Within this study a concept for the investigation of mechanically loaded MEMS materials on an atomic level is introduced, combining high-resolution X-ray diffraction (HRXRD) measurements with finite element analysis (FEA) and mechanical testing. *In situ* HRXRD measurements were performed on tensile loaded single crystal silicon (SCSi) specimens by means of profile scans and reciprocal space mapping (RSM) on symmetrical (004) and (440) reflections. A comprehensive evaluation of the rather complex XRD patterns and features was enabled by the correlation of measured with simulated, ‘theoretical’ patterns. Latter were calculated by a specifically developed, simple and fast approach on the basis of continuum mechanical relations. Qualitative and quantitative analysis confirmed the admissibility and accuracy of the presented method. In this context [001] Poisson’s ratio was determined providing an error of less than 1.5% with respect to analytical prediction. Consequently, the introduced procedure contributes to further going investigations of weak scattering being related to strain and defects in crystalline structures and therefore supports investigations on materials and devices failure mechanisms.

## Graphical abstract

**Figure:**
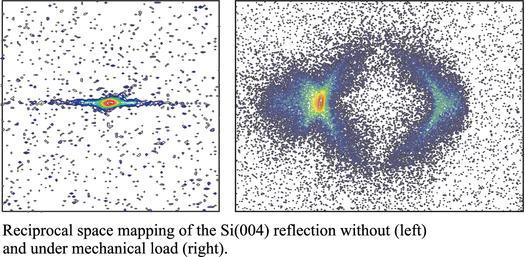

## 1. Introduction

Microelectromechanical systems engineering involves complex design, manufacturing and packaging processes. Owing to specific advantages in functionality and performance, microelectromechanical systems (MEMS) such as sensors, actuators and electronic devices find applications in areas of aerospace, medical, automotive or watch industries, and require high reliability standards. Single crystal materials, especially single crystal silicon (SCSi), are preferentially used in MEMS technology as they present high resistance against aging. Disturbing the crystalline perfection by defects or prestraining influences their mechanical properties as well as their aging behavior. Reduced lifetime and limited reliability are affected by prestrains and damages introduced by large loads during device manufacturing or operation that exceed the critical strength. Furthermore, typical manufacturing process steps, such as deep reactive ion etching (DRIE), thermal annealing, dicing and bonding, could favor failure as well. The wide range of possible failure sources results in a characteristic large variation of strength values over a component [1]. As a consequence, designing and manufacturing reliable MEMS-devices is a challenging task [2,3], which requires high level quality control and reliability studies on potential failure sources as well as in the knowledge of adequate material properties [4–10].

A common strategy to assess, predict, and optimize the reliability of MEMS devices is the fast and cost-effective generation of empirical fracture data obtained by specific “proof tests” [11]. In this context and with the aim to characterize small-scale and scale-specific mechanical properties of MEMS materials, different micro scale experiments have been developed, including tensile bars [7,12–14], different kinds of bending tests [3,16–18], as well as more complex or fixed–free samples and biaxial flexure plates [1,15]. The mentioned experimental methods are often limited to the detection of mechanical properties or to their application in research because of their complexity and requirements in setups and analysis equipment. For the understanding of failure and in order to improve reliable prediction of the performance of micromechanical systems it is essential to obtain further going information about the stressed material on the atomic scale [4,6]. In this context high-resolution X-ray diffraction (HRXRD) methods are well-established experimental approaches for the experimental determination of local strain, deformations and the analysis of defects.

In the frame of the present study, *in situ* mechanical testing was performed on a high-resolution X-ray diffractometer using specific μm-sized SCSi tensile specimens. Related to the experimentally accessible silicon (100) and (110) lattice planes, the XRD investigation was focused on the symmetrical (004) and the (440) reflections. Mechanical loading and geometrical specifications were observed to have a strong impact on the measured rocking curves (RCs) and reciprocal space maps (RSMs) which became rather complex and difficult to interpret, see also [19]. The interpretation and understanding of the complex scattering profiles related to lattice strain and tilt was decisively supported by finite element analysis (FEA). A simple and fast approach providing the simulation of ‘theoretical’ RSM patterns on the basis of FEA was developed and successfully applied for the comprehensive investigation of structural features within the measured patterns. The measured (004) scans were furthermore evaluated for the corresponding [001] Poisson’s ratio whereat the error of the estimated value with respect to the analytical prediction was found to be smaller than 1.5%.

The introduced method extends previously published procedures [20–27] by means of an accurate and versatile testing setup and it combines standard engineering methods (FEA) and advanced methods from material science (HRXRD). The achieved precision within the measurement and evaluation procedure recommend the presented approach for advanced material characterization. In this context, the novel and less extensive numerical approach based on continuum mechanical relations allows the identification and separation of geometrical and structural effects within detected RSM patterns. Beside the determination of the mechanical properties of materials, it contributes to the analysis and deeper understanding of features within diffraction patterns and supports the investigations of weak scattering, which is related to defects.

In silicon-based devices, such as MEMS, structural defects and strain affect aging [28] and limit applications of these devices. Consequently, there is a need for an advanced investigation and characterization strategy which enables the flexible combination and correlation of specialized experimental and numerical analysis methods with respect to the dedicated application area.

## 2. Experimental details

The experimental part hereafter is divided into three complementary sections describing essential advanced developments needed for this study: Section 2.1 – fabrication of test specimen; Section 2.2 – design and implementation of the mechanical test approach; and Section 2.3 – design of the HRXRD experiment and implementation of the *in situ* measurement strategy.

### 2.1. Fabrication of test specimen

**Figure 1.**
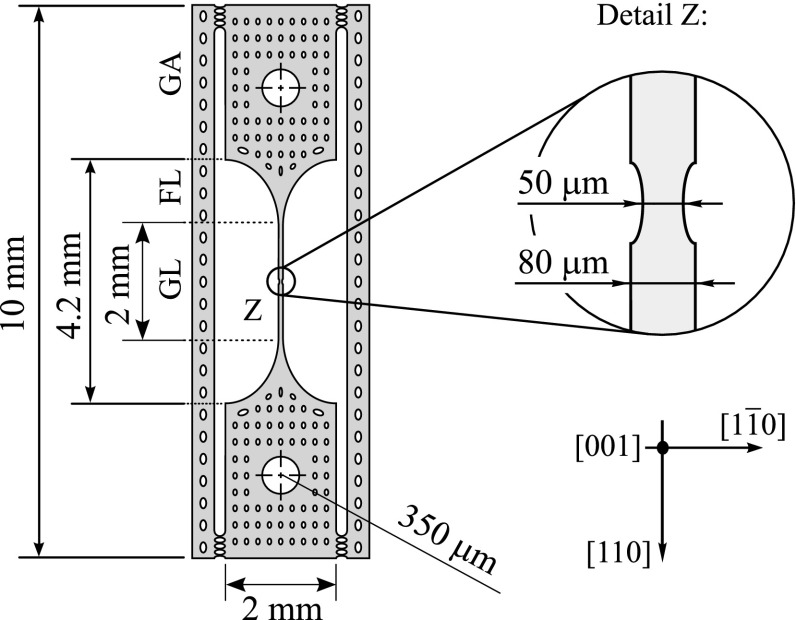
Schematic of tensile specimen of 50 µm thickness including the crystallographic orientations of the silicon crystal. The sample consists of typical domains such as grip area (GA), fillet length (FL) and gauge length (GL). The center of the sample is characterized by a narrowing of the cross-section from 80 µm to 50 µm (Z).

Specific DRIE etched silicon tensile specimens of 10 mm length were fabricated using [001] oriented wafers and elongation was directed along the crystallographic [110] direction. They basically consist of the following parts: two grip areas each with a hole of 0.7 mm in diameter to position adjust and load the sample, a gauge length of 2 mm with a general sample size of 80 × 50 μm, and two stabilization bars for a safe handling which was removed before mechanical loading; cf. Figure 1. The region of maximum loading where fracture preferentially occurs is defined by means of a narrowed cross-section at the center of the SCSi rod towards 50 × 50 μm. The homogeneous strain field within the fillet length is disturbed by this narrowing and the resulting strain field is rather complex. Therefore, enhanced methods such as FEA are required for more detailed investigation and interpretation.

The SCSi specimens were manufactured using SOI (silicon-on-insulator) wafers with a 50 μm thick device layer and standard MEMS manufacturing techniques such as photolithography and DRIE etching processes. Hydrofluoric acid (HF) was used to dissolve the SiO_2_ layer in order to release the specimen from the handle wafer. The samples were extracted one by one in a distilled water bath. A characteristic surface roughness on the etched side-walls was observed and its impact on the fracture strength was investigated elsewhere [29,30].

### 2.2. Design and implementation of the mechanical test approach

**Figure 2.**
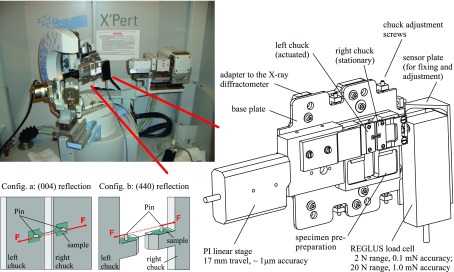
Micro tensile machine consisting of a REGLUS load cell and a standard linear stage (right) form PI (Physik Instrumente). The setup, depicted in the vertical position, can be fixed on a X-ray diffractometer (top left). The front parts of the chucks are replaceable and allow an adaption of the setup with respect to the material orientation of the investigated specimens (down left).

A specific apparatus was developed for the mechanical testing of μm-sized specimens by means of (2-point-) bending experiments and tensile tests; cf. Figure 2. The apparatus basically provides two rigid chucks of which the right, stationary chuck is connected to a load cell (2 N range, 0.1 mN accuracy or 20 N range, 1.0 mN accuracy) and the left, actuated chuck is connected to a standard linear stage (17 mm travel, 1 μm accuracy). The flat design allows the placement of the setup under a microscope and the fixation on the goniometer of a HRXRD diffractometer for *in situ* experiments.

Regarding tensile experiments, the chucks were equipped with pins used for the application of force to the tensile sample. In this configuration only the grip areas are in direct contact with the polished surface of the aluminum chuck while the critical part of the SCSi rod stands free. No clamping or gluing is needed to connect the sample with the testing apparatus and therefore the introduction of unwanted preloading, e.g. prestrain caused by a deadlock, is reduced. A detailed characterization and evaluation of our testing procedure with respect to accuracy and reproducibility was carried out and published previously [31,30].

### 2.3. Design of the HRXRD experiment and implementation of the *in situ* measurement strategy

HRXRD is a non-destructive method for structural characterization of semiconductor materials and systems such as heteroepitaxial, strained layers. Elastic deformation (down to the sub-Angstrom range of inter-atomic distance changes), atomic composition and layer thicknesses can be determined with high accuracy.

The diffraction measurements were performed on a Panalytical MPD high-resolution (PANALYTICAL, Almelo, Nl) diffractometer with CuK radiation selecting the CuKα_1_ line from the X-ray spectrum. RCs were detected using Gutmann optics and a primary four-bounce Bartels Ge (220) monochromator. For RSMs a secondary three-bounce Ge (220) single-crystal analyzer was placed in the beam path providing a resolution of < 10^−4^ degrees with respect to the characteristic diffraction angles ‘2θ’ and ‘ω’, cf. Figure 2.

The mechanical test setup was directly fixed on the goniometer which consists of three orthogonal translators (x-, y- and z directions) and two orthogonal axis of rotation: tilting (χ) and azimuthal rotation (ϕ) respectively. The precise alignment of setup and specimen within the X-ray beam was ensured by accuracies of the goniometer in translation (10 μm) and rotation (0.01°).

For the present investigation a number of alignments steps were performed in order to assess the desired measurement position. The most loaded critical area at the center of the specimen was determined on the loaded specimen by a sequence of ω-scans (RCs). These measurements were performed at equally spaced positions along the SCSi sample. The center was located by means of a shift and broadening of the assessed RCs with reduced intensity corresponding to higher lattice strain and less irradiated material volume. A comparison by overlaying the measured RCs was used to confirm the symmetric state of deformation and the precise loading of the specimen (cf. Supporting Information).

**Figure 3.**
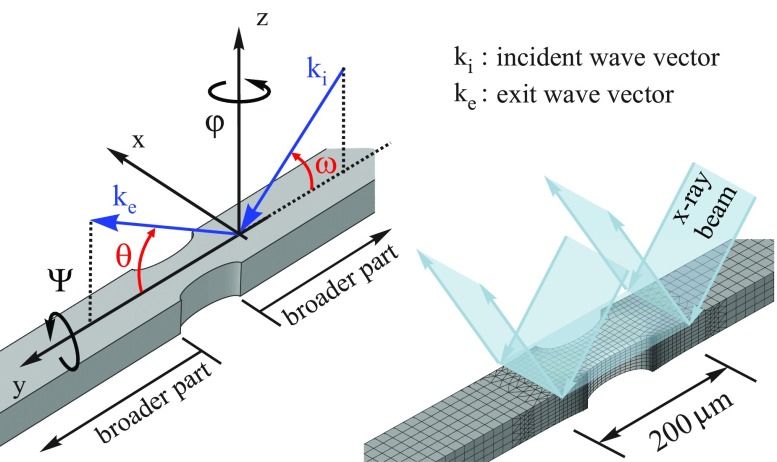
Schematic description of the four scattering angles being relevant for sample alignment and for the measurement of the XRD patterns with respect to setup config. (a), cf. Fig 2.

An incident beam width of 200 μm was used for the measurement of the subsequent RSMs at the specimen center. The adjustment of the mechanically loaded sample within the X-ray beam was performed with respect to maximum scattered intensity which is provided by the particular broader part of the investigated specimen area, cf. Figure 3. This area of maximum intensity was assigned to the theoretical scattering angles, cf. Table 1.

**Table 1.** Characteristic diffraction angles with respect to the measured samples and setup configurations; cf. Figure 2.

Configuration of the mechanical testing setup	Peak angle [°]		
	ω	2θ	ϕ & ψ
Tensile (004), config. (a)	34.5632	69.1264	0 / 90
Tensile (440), config. (b)	53.3508	106.7015	0 / 0

## 3. Analytical and numerical approach

### 3.1. FE simulation

Standard finite element simulation techniques were used for the numerical investigation of the tensile experiment. The entire tensile specimen was modeled with 31,790 second-order hexahedral and 1808 second-order wedge elements. In order to ensure an accurate resolution of the local deformation and stress fields the mesh was refined in the vicinity of the critical cross-section at the center of the specimen, providing at least eight elements over the thickness. SCSi was assessed as an elastic cubic orthotropic material defined by three elastic coefficients C_11_=165.8 GPa, C_12_=63.9 GPa and C_44_=79.6 GPa [32]. As long as the strains are small enough, i.e. do not exceed ~1%, the framework of linear elastic field theory provides a good approximation to the underlying nonlinear behavior.

### 3.2. HRXRD analysis

Two basic types of XRD measurement were carried *in situ* on loaded tensile specimens to record RCs and RSMs. Single scans provide information about crystalline perfection, compositional homogeneity, magnitudes and gradients of elastic strain which become accessible by analyzing the profile shape, width and position [33–37]. RSM provides more detailed information on the microstructure through the separation of complex diffraction features such as tilts and mosaicity into distinct features such as Bragg and diffuse scattering. Diffuse scattering appears as distributed weak scattering in the reciprocal space map and is raised from sample imperfections such as defects and tilt [35,36,38–40]. The small specimen size, the material anisotropy and the aimed assessment of weak scattering require the high performance of a state-of-the-art X-ray diffractometer with very high angular resolution [34,36]. The analysis of the measured patterns was based on Bragg’s law, Equation (1) and its differentiation, Equation (2). These expressions estimate the basic relationship between scattering angle θ and the interatomic spacing *d* of the crystal lattice:

(1) $$n\cdot \lambda =2\cdot \;d_{hkl}\cdot sin\;\theta_{hkl}$$

(2) $$\Delta \theta =\frac{\Delta d}{d}\cdot tan\theta =-\epsilon \cdot tan\theta$$

Consequently, lattice strain in terms of *ε* = *Δd/d* is estimated by the variation of the scattering angle *Δθ.*

### 3.3. Simulation of RSM pattern

**Figure 4.**
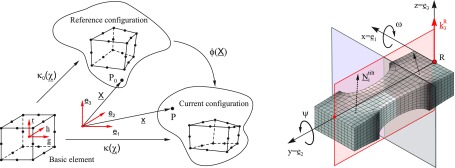
Schematic of mapping and relations between the different states of the elements in the FE simulation with respect to a 20 node quadratic brick element. Indexes (represent the coordinate system used in the FE simulation. The figure depicts furthermore the investigated central part and symmetry planes of the tensile specimens as well as the definitions of the quantities used for the introduced simulation method of RSM patterns.

Each diffraction spot in an RSM pattern (reciprocal lattice point) represents the orientation and spacing of a set of lattice planes in the crystal. Hence, the shape and features of RSM patterns are affected by lattice strain and tilt caused by mechanical or thermal loading [34]. Related to the FE calculations these sets of lattice (diffraction) planes were assumed to be represented by single elements and element nodes which provide the mentioned information about d-spacing and lattice tilt and consequently silicon strain and orientation. The main steps for the simulation of RSM patterns and determination of the required quantities based on nodal coordinates of the applied FE mesh are presented below:

First, elements and nodes are regarded in three different continuum mechanical configurations, cf*.* Figure 4, whereas the initial configuration represents the basic state of the element described by the isoparametric element coordinates (*g*,*h*,*r*) [42,41]. The ‘reference configuration’ represents the element shapes of the unloaded FE mesh and the ‘current configuration’ represents the calculated deformed element shapes after loading. The positions of any material body points represented by finite element nodes within reference and current configuration are assessable by the position vectors $\underline{X}=X_{i}\cdot {\underline{e}}_{i}$ and $\underline{x}=x_{i}\cdot {\underline{e}}_{i}$ defined with respect to a standard basis $\epsilon =\{{\underline{e}}_{1},{\underline{e}}_{2},{\underline{e}}_{3}\}$. *X*_*i*_ and *x*_*i*_ represent the so-called material and spatial nodal coordinates. The linking of the configurations is either provided by deformation gradients or mapping functions applied on the particular finite elements.

Second, at any time *t*, the deformation of a finite element is describable by nodal positions and the underlying element specific shape functions [42,41]. Given this fact, *κ*_0_ $\left(\underline{\chi }\right)$ and *κ* $\left(\underline{\chi }\right)$ maps were defined providing a linking from the basic element state to the reference and current configuration respectively, cf*.* Equation (3).

$$\kappa_{0}(\underline{\chi })=M(\underline{\chi })\cdot \underline{\hat{X}}$$

(3) $$\kappa (\underline{\chi })=M(\underline{\chi })\cdot \underline{\hat{x}}$$

$M(\underline{\chi })$ corresponds to a matrix consisting of the isoparametric representation of the shape functions expressed in coordinates (*g*,*h*,*r*) and denoted as $\underline{\chi }$. The position vectors $\underline{\hat{X}}$ and $\underline{\hat{x}}$ are related to the reference and current configuration respectively denoting element nodal coordinates provided prior and after the FE calculation. Based on Equation (3), a mapping function $\phi \;(\underline{\chi })$ from reference to current configuration was derived by the composition operation (∘) $\kappa_{0}\left(\underline{\chi }\right)$ and $\kappa (\underline{\chi })$; cf. Equation (4).

(4) $$\phi \;\left(\underline{\chi }\right)=\kappa \circ \kappa_{0}^{-1}\left(\underline{X}\right)$$

Third, the calculation of strain *ε*_*i*_(*t*) and orientation for every element node *i* at the time *t* in the reference and current configuration is provided by the deformation gradient which is classically defined as $F=\partial \underline{x}/\partial \underline{X}$. Within the framework of this approach and related to a mapping function $\phi \;(\underline{\chi })$ the deformation gradient can be written as follows, cf. Equation (5):

(5) $$F=\frac{\partial \underline{x}}{\partial \underline{X}}=\frac{\partial \phi (\underline{\chi })}{\partial \underline{X}}$$

Equation (6) provides a compact expression for the deformation gradient by the combination of Equations (3)–(5):

$$F=\frac{\partial \phi \;(\underline{\chi })}{\partial \underline{X}}=\frac{\partial \kappa }{\partial \underline{\chi }}\cdot \frac{\partial \kappa_{0}}{\partial \underline{X}}={\left[\frac{\partial M\;(\underline{\chi })}{\partial \underline{\chi }}\right]}^{T}\cdot \underline{\hat{x}}\cdot {\left({\left[\frac{\partial M\;(\underline{\chi })}{\partial \underline{\chi }}\right]}^{T}\cdot \underline{\hat{X}}\right)}^{-1}$$

(6) $$F=\left(A\cdot \underline{\hat{x}}\right)\cdot \left(A\cdot \underline{\hat{X}}\right)$$

In this context ***A*** is a (*n x n x n*) matrix containing the derivatives of the element shape functions with respect to isoparametric coordinates $\underline{\chi }=(g$,*h*,*r*). Using well defined transformation rules, strain *ε*_*i*_(*t*) and orientation, denoted as ${\underline{N}}_{i}^{tilt}\left(t\right)$, are evaluable for every element node *i* at the time *t* with respect to the element surface normal ${\underline{\overset{ˇ}{e}}}_{3}\left(t\right)$ of the undeformed element in the reference configuration; Equations (7–9):

(7) $${\underline{N}}_{i}^{strain}\left(t\right)=F_{i}\left(t\right)\cdot {\underline{\overset{ˇ}{e}}}_{3}\left(t\right)$$

(8) $$\varepsilon_{i}\left(\text{t}\right)\;=norm\left({\underline{N}}_{i}^{strain}\left(t\right)\right)-1\;$$

(9) $${\underline{N}}_{i}^{tilt}\left(t\right)=F_{i}\left(t\right)\cdot {\underline{\overset{ˇ}{e}}}_{3}\left(t\right)$$

Fourth, according to the measurement procedure, the theoretical RSM pattern was determined by means of calculated deviation of strain and orientation (tilt) with respect to reference values derived at the border of the investigated area of the specimen; cf*.* Figure 4. Reference strain $\varepsilon_{R}\left(t\right)$ and orientation ${\underline{k}}_{3}^{R}\left(t\right)$ were assessed by averaging strain and element surface normal around a reference point *R* corresponding to the ‘origin’ in the simulated and measured reciprocal space maps. In this context, every element node contributes a data point ($\Delta \varepsilon_{i}\left(t\right)$,α) to the RSM pattern representing one specific lattice spacing; cf*.* Equation (10), and lattice tilt; cf. Equation (11).

The relation between the searched diffraction angle *θ* and lattice spacing is provided by Equation (2). For reason of visibility and interpretation, every data point was assigned with an estimated intensity distribution corresponding to a Gaussian intensity profile *f(r,s)*; cf*.* Equation (12). The overlaying and summation over the contribution of all data points provided an intensity distribution which is proportional to the number of data points in a certain area of the RSM pattern.

(10) $$\Delta \varepsilon_{i}\left(t\right)\;=\varepsilon_{i}\left(t\right)-\varepsilon_{R}\left(t\right)$$

(11) $$\alpha =cos^{-1}\left(\frac{{\underline{N}}_{i}^{tilt}\left(t\right)\cdot {\underline{k}}_{3}^{R}\left(t\right)}{\left|{\underline{N}}_{i}^{tilt}\left(t\right)\right|\cdot \left|{\underline{k}}_{3}^{R}\left(t\right)\right|}\right)\;$$

(12) $$f\left(r,s\right)=\text{exp}\left(\frac{-r^{2}}{2\cdot s^{2}}\right)\;$$

## 4. Results and discussion

### 4.1. Experimental HRXRD patterns

Reference measurements at the central part of an unloaded specimen were performed in order to assess and to exclude potential impacts related to defects and pre-strains in the material, e.g. introduced during manufacturing. Both the RC and RSM patterns confirmed by their typical size and shape the expected high quality of the silicon single crystal. The full width at half maximum corresponded to a value of FWHM=0.0040°; cf*.* Figure 5(a).

**Figure 5a.**
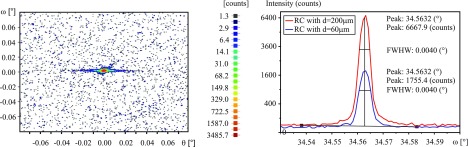
Reference measurements at the center of an unloaded tensile specimen. Left: RSM of the Si(004) reflection. Right: RCs of the Si(004) reflection measured with a X-ray beam width of d=60 μm and d=200 μm respectively.

Several RSM measurements were performed at the critical central part of the tensile specimen which was loaded between 1 N and 6 N respectively. Influenced by the mechanical load, the RSM patterns become quickly rather complex. Representative for all performed measurements, in the following the RSM patterns, FE calculations as well as simulated patterns are discussed for an axial load of 4 N. However, a change in load mainly resulted in a shrinking (decreasing load) or expansion (increasing load) of the features and the shapes in general; cf*.* Figure 5(b) and Supporting Information. Reciprocal space maps were assessed on the (004) and (440) reflections provided by the illustrated setup configurations (a) and (b) of Figure 2. The detected patterns were depicted with a logarithmic intensity scaling; cf*.* Figure 5(b).

**Figure 5b.**
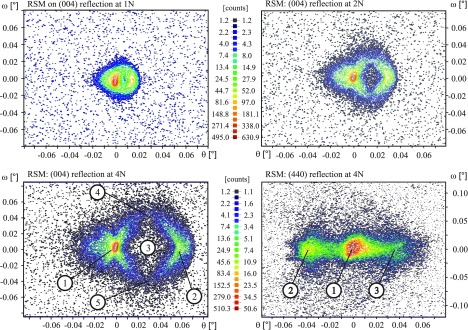
Top: Selected RSM patterns on the (004) reflection visualizing the impact of tensile loading at 2 N (left) and 3 N (right) tensile force application. Bottom: RSM pattern on the (004) (left) and (440) (right) reflections at an axial load of 4 N.

The point of maximum intensity which corresponds to the point of the sample alignment within the diffraction plane was located at the origin (0,0). According to the symmetry of the specimen and intrinsic symmetric state of loading at the investigated center part of the specimen, symmetries are recognizable within the patterns. In Figure 5(b), the (004) pattern is characterized by five main features assigned with markers ① – ⑤. A symmetrical arrangement of the measured shape is obtained with respect to the horizontal axis (±ω): there are two delimited areas with higher intensity values which are horizontally aligned and connected by two curved tails enclosing a ‘hole’-like section. Consequentially, a symmetric expansion of the features was observed as wells under increased loading of the specimen whereat the ‘hole’ became visible for a first time as such at a load of 2 N.

According to Equation (2), features ① and ② indicate two areas of parallel oriented but differently strained lattice spacings. Related to the position and intensity values, feature ② points to the thinnest and most strained part of the tensile sample whereas feature ① corresponds to the thicker and therefore less strained area. These features were confirmed and investigated by additional ω/2θ-scans; cf*.* Supporting Information. Beside lattice strain, lattice tilt is also observed from RSM patterns. Maximum tilt characterized by a significant drift in ω-direction of the pattern is located in the middle between the two points of maximum intensity at the particular turning points of the tails, cf*.* Figure 5(b) markers ④ and ⑤. Considering the slight offset of the measured RSM with respect to the horizontal axis, lattice tilt is assessed at the estimated center and outer apex of the tail-like features by means of their shift within ω; cf*.* Table 2.

**Table 2.** Estimation of lattice tilt at the tail-like features ④ and ⑤ within the (004) RSM patterns of Figure 5(b). Indexes ‘C’ and ‘A’ correspond to the estimated center and apex of the tails. Tilt was determined for the upper ④ and bottom ⑤ tail by means of the offset corrected measurement values represented by the particular absolute values |tilt_C_| and |tilt_A_|.

Features	Measured values					Offset corrected	
	Force	2θ	ω_C_	ω_A_	offset	|tilt_C_|	|tilt_A_|
	[N]	[°]	[°]	[°]	[°]	[°]	[°]
④			0.0238	0.0309		0.017	0.024
	1.042	69.1408			0.0070		
⑤			−0.0095	−0.0172		0.017	0.024
	
④			0.0263	0.0385		0.025	0.038
	2.043	69.1552			0.0009		
⑤			−0.0248	−0.0368		0.026	0.038
	
④			0.0436	0.0576		0.040	0.054
		69.1829			0.0035		
⑤	4.038		−0.0365	−0.0506		0.040	0.054
	

The (440) pattern is characterized by three circular spots of higher intensity horizontally arranged, which are equally spaced and connected with a band-shaped intensity distribution. Furthermore, a symmetry of the pattern with respect to the horizontal axis (±ω) representing the silicon lattice tilt parameter is also recognizable. The mainfeatures ①, ② and ③ indicate areas of three parallel but differently strained silicon crystal lattice parameters. The expansion and weak scattering in ω direction which become mainly pronounced and recognizable at position ② and ③ show furthermore a certain amount of tilt within the lattices. This is probably caused by a small misalignment of the specimen. In comparison with the (004) pattern, the overall intensity was found to be smaller by a factor of 10.

**Figure 6.**
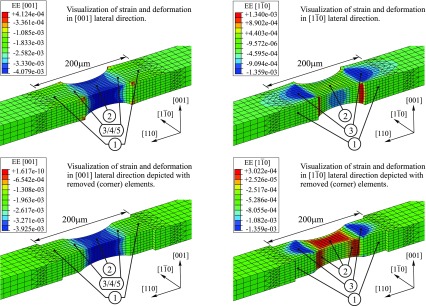
Strain field corresponding to the (004) and (440) RSM pattern including a scaling factor of 100 and 60 for the deformation into lateral [110] direction and [001] direction respectively. Essential but underlying parts of the strain field were enhanced by removing the outer elements in the 200 µm irradiated area and the disturbing corners at the central zone.

A first qualitative interpretation and correlation between the measured RSM patterns and the complex state of structural deformation at the investigated center of the loaded specimen is provided by FEA. An overview on the calculated lateral strains is depicted in Figure 6. The deformation in the [110] and [001] directions was scaled by factors of 100 and 60 respectively providing a better visualization and comprehension. Specific characteristics of the deformed specimen were made clearer by the removal of selected elements within the 200 μm irradiated area which included e.g. the highly distorted corners at the central zone; cf*.* Figure 6. Conforming with the identified features in the patterns related to the lattice spacing, the corresponding areas in the calculated strain field were highlighted with the markers ① – ⑤. The origin of the hole-like feature within the (004) pattern can be understood by analyzing the deformations in the transition area between the parallel broader parts ① and the central part ② of the specimen. Following the progress of the surface normal along the center line from ① to ②, the point of maximum tilt is located in the vicinity of markers ③ – ⑤ providing maximum shift with respect to the reference (alignment) area ①. Consequently, the ‘hole’ is explainable by the combination and interaction of changes in lattice tilt and lattice spacing between ① and ②.

### 4.2. Simulated patterns

**Figure 7.**
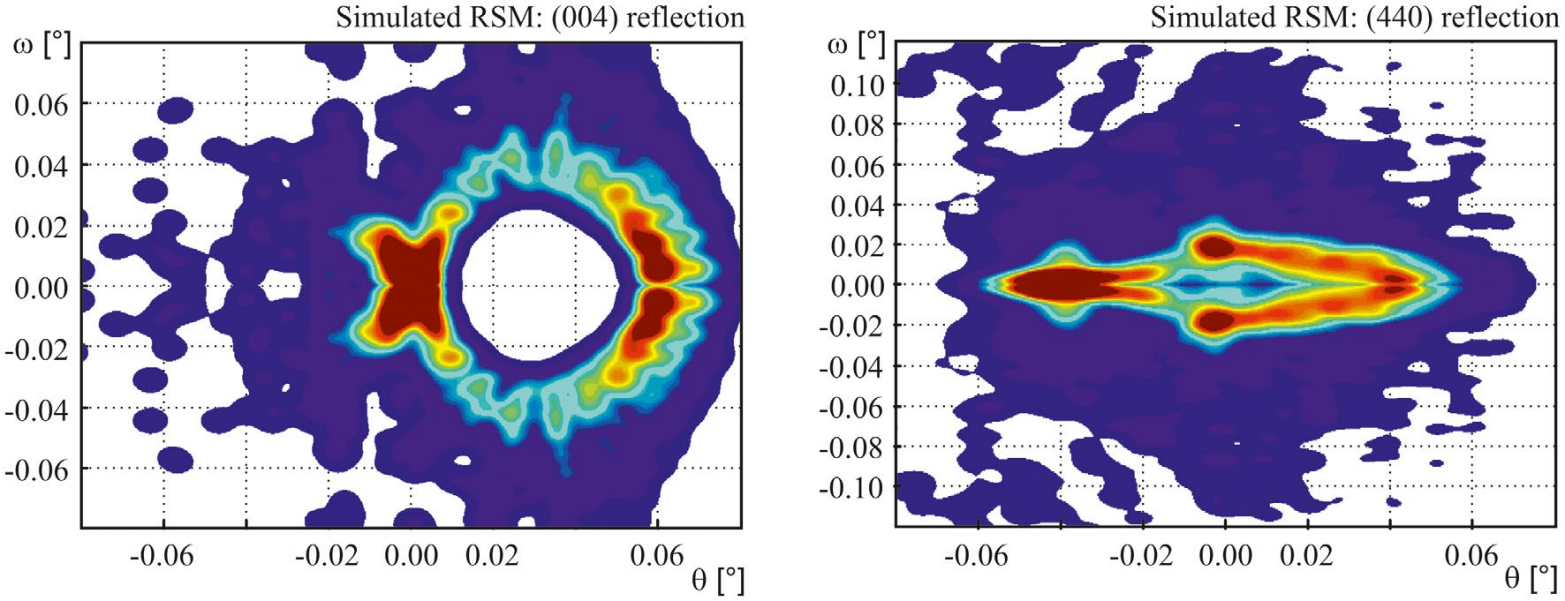
Simulated RSM patterns of the (004) and (440) peak respectively based on the deformation field obtained by FE calculation at a load of 4 N. The simulation was based on the particular outer element layer of the irradiated area. The colors correspond to the normalized intensity concentration derived by the applied exponential energy distribution.

An enhanced verification of the qualitative evaluation above as well as of the mentioned structural impact on the measured RSM patterns was provided by the simulated patterns depicted in Figure 7. The estimated intensity values were normalized to 1 and were obtained by the application of the exponential distribution function to each data point. Dependent on the applied parameters of the estimated exponential energy function, specific features and details of the pattern become more pronounced or ‘washed out’ respectively.

Related to the analytical focus of the investigation, a depiction by single data points was found to be more effective for the identification, assignment and separation of structural features which might be excluded for further evaluation considering the overall specifications of the HRXRD setup.

Furthermore, this approach is the basis for ongoing investigation of the measured patterns with respect to weak scattering, e.g. caused by defects. A collection of simulated patterns based on various numbers of elements and with different energy settings as well are provided in the Supporting Information.

The depicted intensity distribution in Figure 7 is based on the mentioned simplified estimation according to Equation (2) considering the equally weighted contribution of all calculated (depicted) nodes. Specific impacts on the intensity such as penetration depth of the X-ray beam and related scattering effects are not assessed with the presented approach and therefore the coloring between measurement and simulation is not directly comparable.

In the case of the (004) pattern, a very good agreement of the simulation with the measurement results was realized. Only the top layer of elements, corresponding to a material thickness of 6.25 μm, was used for the simulation. The mentioned limitation of the applied intensity estimation which is only based on the particular node position and orientation is reflected in the simulated (440) pattern. The main features were recognizable as well while in comparison with the measured pattern the simulated pattern appears broader and more blurred, with respect to its expansion in ω direction. In this context, the change of the specimen geometry in the central zone along the [110] direction is only partly assessed in the simulation. This shift of the scattering plane towards the notch root is responsible for the weaker feature ③ within the (440) pattern, cf*.* Figure 5. A better correlation can be realized by a refinement of the discretization within the FE model. The combination of the present simplified approach with more sophisticated methods for the analysis of the scattering process such as dynamical scattering theory is required for an improved agreement between simulated and measured patterns.

Taking into account the mentioned limitations, a quantitative strain investigation of the measured and simulated pattern was performed with respect to the central part of specimen. Table 3 provides an overview of the determined strain parameters. The corresponding values of the FE simulation $\Delta \varepsilon_{lateral}^{FEA}$ were calculated as the averaged nodal values which contribute to the maximum intensity in the simulated pattern. The estimated deviation between simulation $\Delta \varepsilon_{lateral}^{FEA}$ and measurement $\Delta \varepsilon_{lateral}^{RSM}$ for both patterns was found between 0.6% and 3.5%.

**Table 3.** Determined strain values from measured and simulated RSM patterns.

Features		Force	2θ	$\Delta \varepsilon_{lateral}^{RSM}$	$\Delta \varepsilon_{lateral}^{FEM}$
		[N]	[°]	[-]	[-]
Tensile(004)	①	4.038	69.1264	0.148(1)	0.150(2)
	②		69.2432		
Tensile(440)	①	4.023	106.6232	0.051(1)	0.050(2)
	②		106.7016		
	③		106.7832	0.053(1)	0.053(2)

### 4.3. Poisson’s ratio

**Figure 8.**
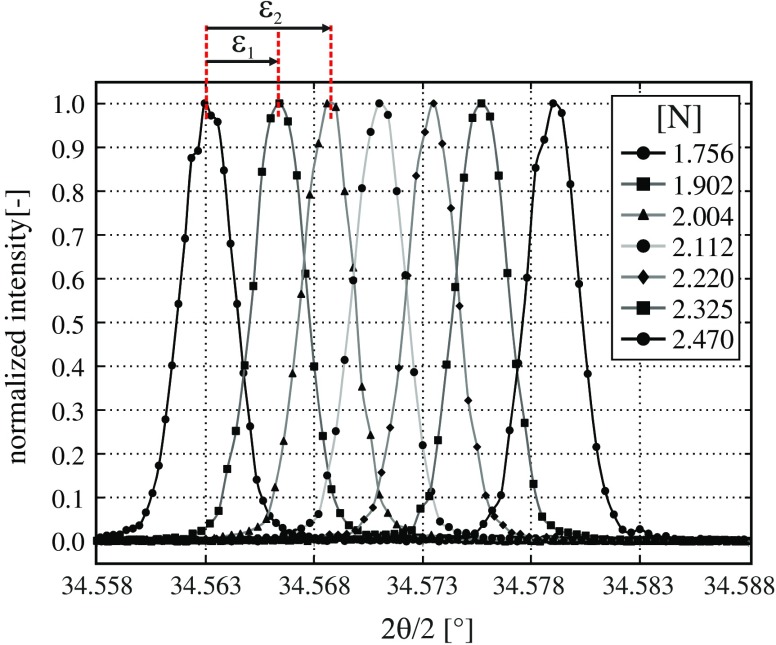
2θ/ω scan on the (004) reflection detected at the increased loaded gauge region of the tensile specimen.

The measurement setup enables furthermore the determination of Poisson’s ratio which is defined as the ratio between lateral and axial strain. In this context, several 2θ/ω scans were measured at the broader gauge region of a sequentially loaded specimen providing the required homogeneous uniaxial strain field. Starting position and ‘reference state’ of the analysis was a specimen which was submitted to an initial loading of 1.756 N. After adjusting this sample within the diffractometer the initial state was assigned to the theoretical scattering angles of Table 1. The measured scans on the (004) reflection are characterized by an expected shift of the peak position with increasing loads towards higher 2θ angles indicating a decrease of the lattice spacing; cf*.* Figure 8.

An overview of the determined peak position, calculated strain values and of the derived Poisson ratio is provided in Table 4. The out of plane strain *ε*_*lateral*_ was calculated using Bragg’s equation in combination with the measured peak quantities; cf*.* Equation (2). An experimental assessment of the axial strain is provided by the setup as well, e.g. by means of optical methods such as microscopy [31,45]. In the frame of the present work axial strain was estimated by FE calculation providing a specimen related expression denoted as *ε*_*axial.th*_ which is dependent on the applied force *F* expressed in [N]: $\varepsilon_{axial.th}\left(F\right)=0.00153\cdot F-0.000007$. The averaged Poisson’s ratio over all measurements was finally calculated as ν=0.366 with a relative error of 1.38% on the theoretical value [43,44].

**Table 4.** Overview on the evaluated XRD profile scans of Figure 8 in terms of peak position and offset, force, strain and Poisson’s ratio. The required calculations for *ε*_*axial*_ and *ε*_*lateral*_ were exemplary performed on the directly bordering peak or evaluation positions.

	Measured values				Calculated values			
2θ/ω Scan	Force	Peak 2θ	Peak ω	offset (ω)	ε_axial.th_	ε_axial_	ε_lateral_	Poisson’s
	[N]	[°]	[°]	[°]	[–]	[–]	[–]	ratio
Ref	1.756	69.1262	34.5632	0	0.002680	0	0	—
1	1.902	69.1328	34.5611	0,0053	0.002903	0.000223	8.36E-05	0.374
2	2.004	69.1372	34.5593	0,0093	0.003059	0.000156	5.57E-05	0.357
3	2.112	69.1420	34.5582	0,0128	0.003224	0.000165	6.08E-05	0.368
4	2.220	69.1468	34.5574	0,0161	0.003390	0.000165	6.08E-05	0.368
5	2.325	69.1514	34.5561	0,0196	0.003550	0.000161	5.82E-05	0.363
6	2.470	69.1580	34.5545	0,0245	0.003772	0.000222	8.36E-05	0.377

Both the qualitative and quantitative results confirm a good agreement between simulation and measurement, and therefore the accuracy and potential of the introduced method for further ongoing investigations based on precisely loaded specimen related to weak scattering, the behavior of defects and material research in general.

## 5. Conclusions

Mechanical and structural properties are correlated, especially for MEMS. Therefore, a new concept for *in situ* testing of MEMS parts is needed which is combining mechanical testing and structural analysis down to atomic resolution using HRXRD experiments and FE simulations for both mechanical behavior and multidimensional X-ray pattern reconstruction. The complementarity of methods allows increased understanding of the mechanical behavior of MEMS based on structural specifications and modifications during loading. *In situ* HRXRD measurements such as rocking curves (RCs) and reciprocal space maps (RSMs) on loaded μm-sized SCSi tensile specimen were performed and analyzed for lattice strain and lattice tilt. Loading and geometrical specifications, e.g. the reduction of the cross-section at the center part of the samples, have a strong impact on the measured (004) and (440) RSM patterns which resulted in complex X-ray scattering features. FE simulations support the understanding and interpretation of the observed features within the detected RSMs. Additionally, theoretical RSM patterns were simulated based on the complex state of deformation at the mechanically loaded structure and qualitatively as well as quantitatively successfully evaluated for lattice strain and lattice tilt. Related to specific features of the patterns, estimated relative errors of 0.6–3.5% between simulation and measurement were calculated, which confirmed the observed good visual agreement of the RSM patterns for the tensile experiments. In addition, Poisson’s ratio along the [001] direction was determined providing a relative error of 1.4% with respect to theoretical values. The results confirm the accuracy of the testing setup and complement the possibilities shown for the determination of material properties and characterization of materials in general [29,30].

The introduced method provides – in comparison to other techniques [20–27] – an efficient and easy to handle approach for the investigation of HRXRD patterns considering arbitrary loaded structures. It presents the combination of standard engineering methods (FEA) with standard methods from material science, allowing investigations on an atomic level (HRXRD). The evaluation and interpretation of RSM patterns with respect to deviations from crystal perfection requires a proper separation of geometrical and structural effects. The presented concept contributes to ongoing investigations of materials and components with respect to defects and related failure modes in crystalline structures and will lead to a better understanding of aging and degradation modes, which is a necessity for the future of MEMS.

## Disclosure statement

No potential conflict of interest was reported by the authors.

## Supplemental data

The supplemental material for this paper is available online at http://dx.doi.org/10.1080/14686996.2017.1282800

## Supplementary Material

Supporting-Information_2017-01-11.pdf
