# Supplementary material for: In situ MEMS testing: correlation of high-resolution X-ray diffraction with mechanical experiments and finite element analysis
Source: Sci Technol Adv Mater. 2017 Mar 31;18(1):219–30. doi: 10.1080/14686996.2017.1282800 (PMC5425911; doi:10.1080/14686996.2017.1282800)
Supplement: Supporting-Information_2017-01-11.pdf [file tsta_a_1282800_sm8147.pdf]

Supporting Information for

In situ MEMS testing: correlation of high-resolution X-ray diffraction with mechanical experiments and finite element analysis

Andreas Schifferle*,1, Alex Dommann2 and Antonia Neels2

[1] Helbling Technik AG, Schachenallee 29, 5000 Aarau, Switzerland
[2] Empa, Überlandstrasse 129, 8600 Dübendorf, Switzerland

Keywords high-resolution X-ray diffraction (HRXRD), reciprocal space mapping, in situ material characterization, single crystal silicon, finite element analysis (FEA)

* Corresponding author: andreas.schifferle@helbling.ch

A. Alignment of the tensile specimen and accuracy estimation

The remarks hereafter were made with respect to the (004) reflection. Text and figures are taken from [42].

After mounting the mechanical test setup on the goniometer, placing and loading of the specimen the zone with the reduced cross sectional dimension was detected by a sequence of Rocking Curves (RCs) detected along the SCSi specimen. The spacing between the single measurement positions was set to 10μm. Within these measurements the incident X-ray beam was limited to a line width of 60μm by adjusting the corresponding slits in the beam path, *cf.* Fig. S1. The alignment of the sample in the X-ray beam with respect ω, φ and χ was done on the broader part of the SCSi specimen labelled as 'Pos1' and 'Pos5', *cf.* Fig. S1. The corresponding peak was named 'alignment peak' and was assigned with the nominal diffraction angle, *cf.* Table 1.

A representative set of measured Si(004) RC in the vicinity of the specimen center is depicted and evaluated with respect to the different zones on the test specimen in Fig. S1b. RCs measured at 'Pos1' and 'Pos5' are characterized by one single reflection whereas the RCs 'Pos2' and 'Pos4' show an additional second peak. One reflection remains located at 34.5632° and corresponds to the alignment (nominal) peak. The second one is set on its right indicating a decrease in lattice spacing. Furthermore it is characterized by a peak broadening and reduced intensity. Both findings are indicators for an increase in lattice tilt and a reduction of the irradiated material volume respectively.

The center of the specimen (Pos 3) was finally recognized by the disappearance of the alignment peak at 34.5632°. The symmetric state of deformation and the precise loading of the specimen were finally confirmed by the congruence of the measured curves from 'Pos 1' with 'Pos 5' as well as those from 'Pos 2' with 'Pos 4', *cf.* Fig S1b.

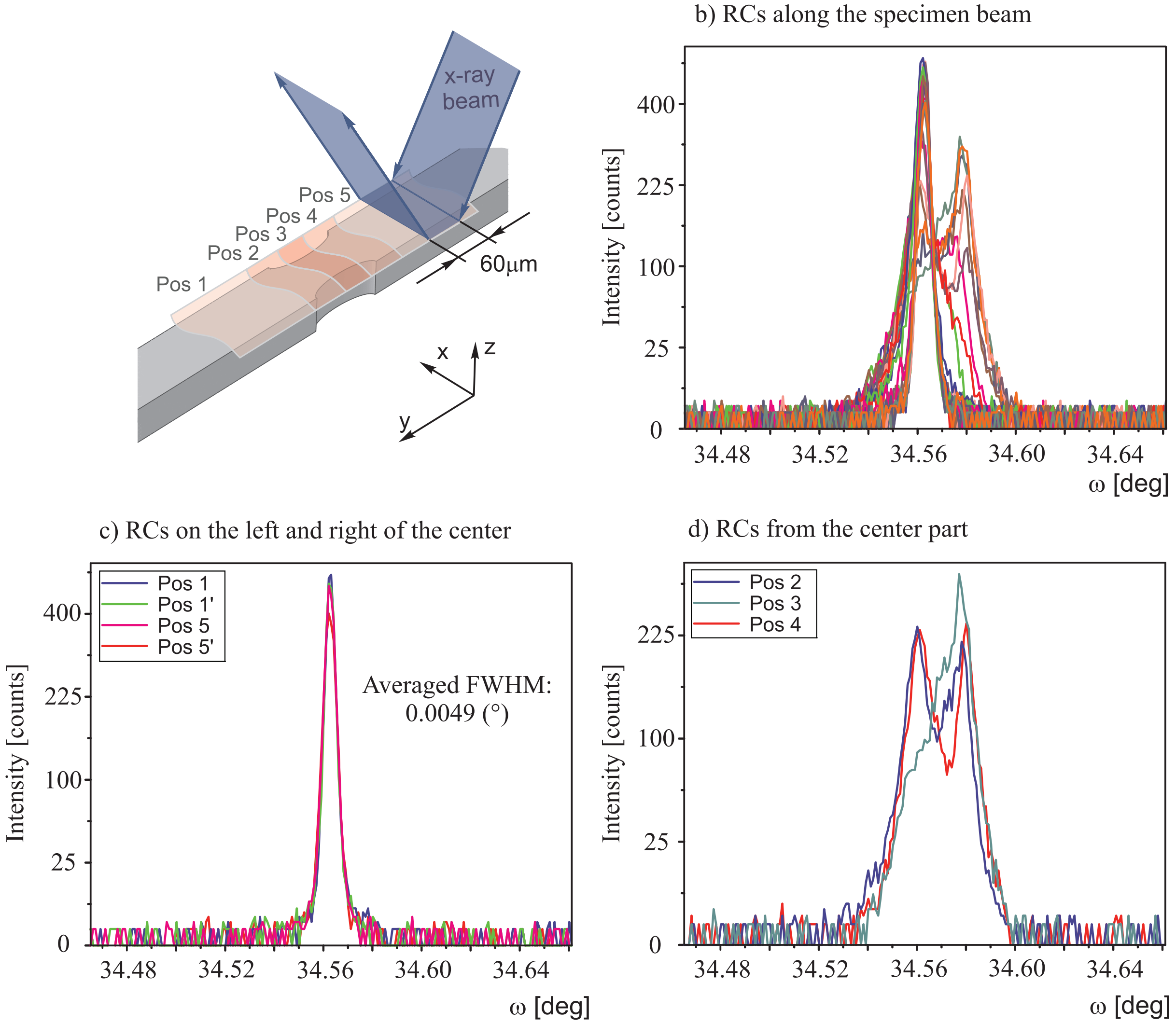

Figure S1. a) Schematic depiction of the Rocking Curve (RC) measurement on the (004) reflection along the y-axis of a tensile specimen which is loaded with 1N. b) The whole set of measured RCs which is separated in the contribution parts of different positions on the specimen. The RCs confirm the very precise alignment of the sample distinguishable by an almost perfect overlapping of measured curves before and after the central region. c) The difference in lattice spacing in the center of the loaded specimen with respect to the region of the alignment leads to an additional peak on the right side of the alignment peak at 34.5632° (nominal value).

B. HRXRD-Measurements: Si(004) RSM pattern

The impact of mechanical loading on the RSM patterns in general as well as on specific features was assessed by means of sequentially loaded specimens. Additional 2θ/ω scans confirm furthermore the highlighted main features by means of two peaks separate by a ‘hole. The presented figures of are taken from [42].

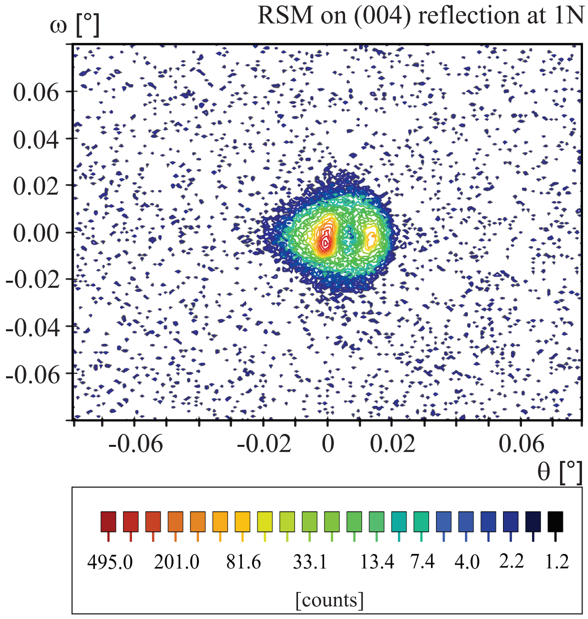

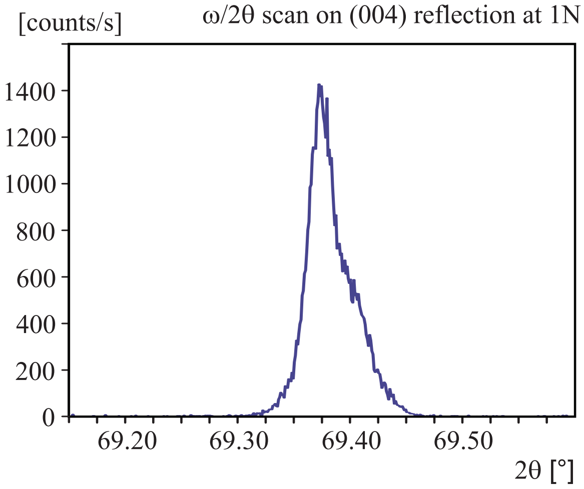

- RSM measurement time: 12h.
- The scan was measured through the center of the RSM at ω=0.

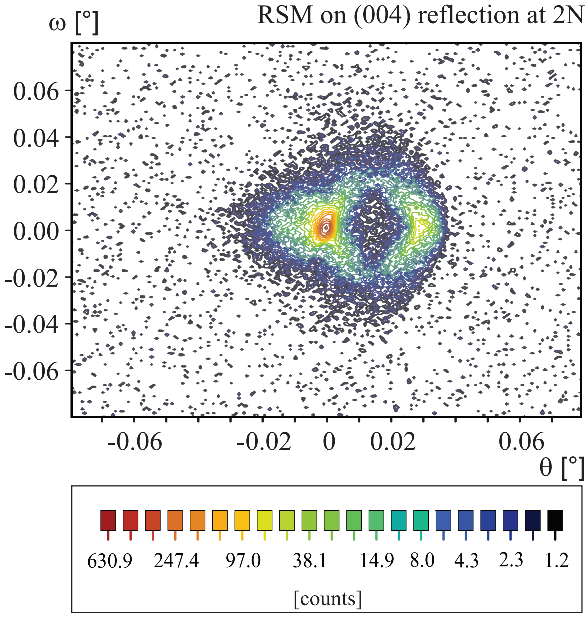

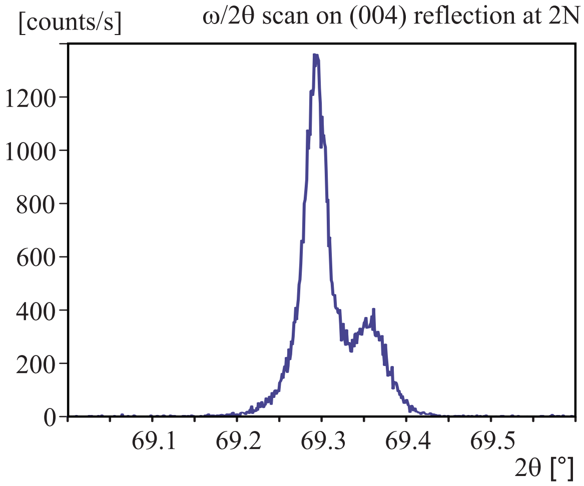

- RSM measurement time: 12h.
- The scan was measured through the center of the RSM at ω=0.

RSM on (004) reflection at 3N

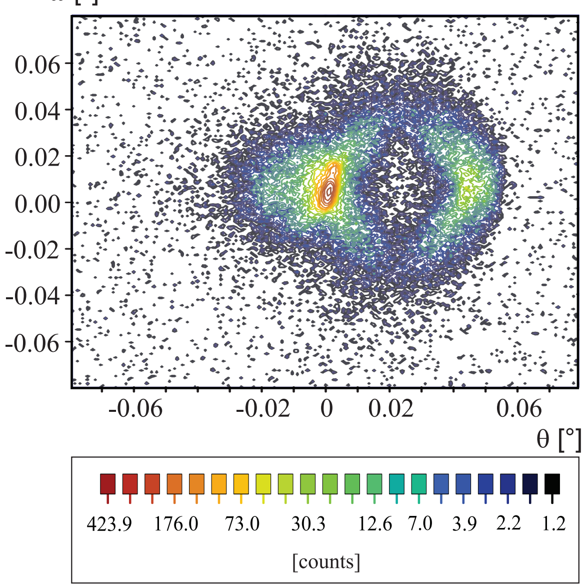

ω/2θ scan on (004) reflection at 3N

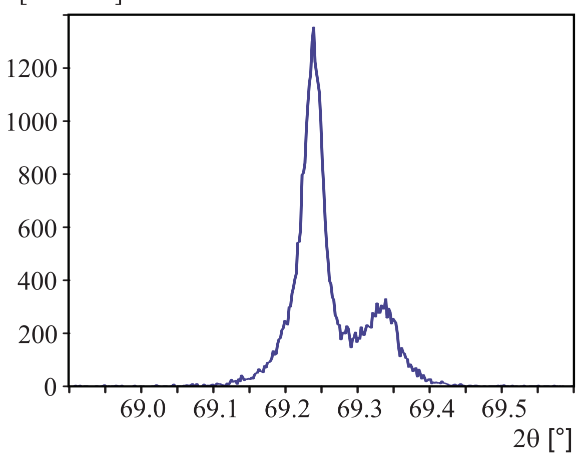

- RSM measurement time: 12h.
- The scan was measured through the center of the RSM at ω=0.

RSM on (004) reflection at 4N

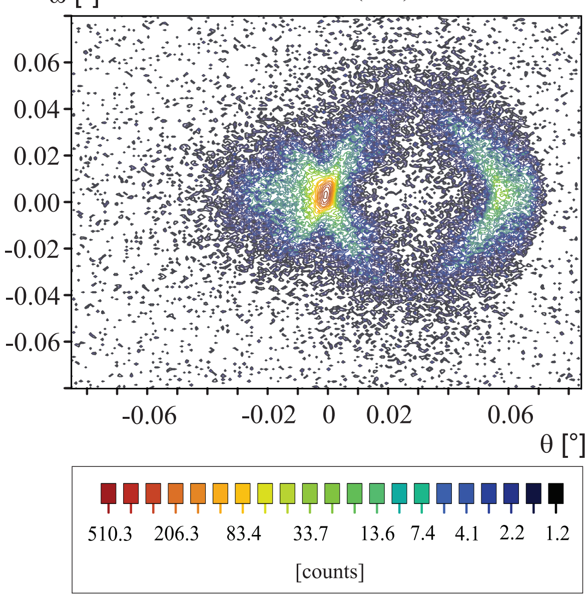

ω/2θ scan on (004) reflection at 4N

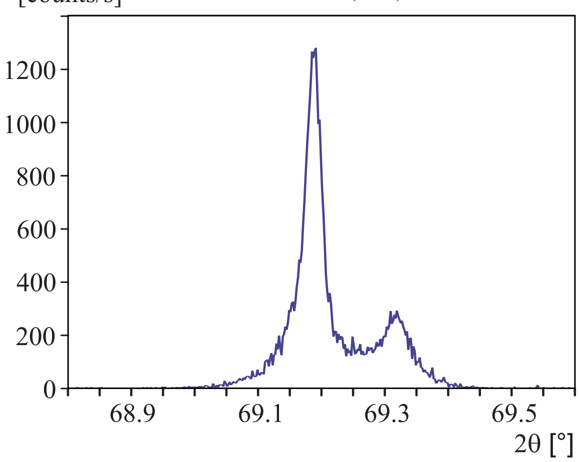

- RSM measurement time: 12h.
- The scan was measured through the center of the RSM at ω=0.

RSM on (004) reflection at 5N

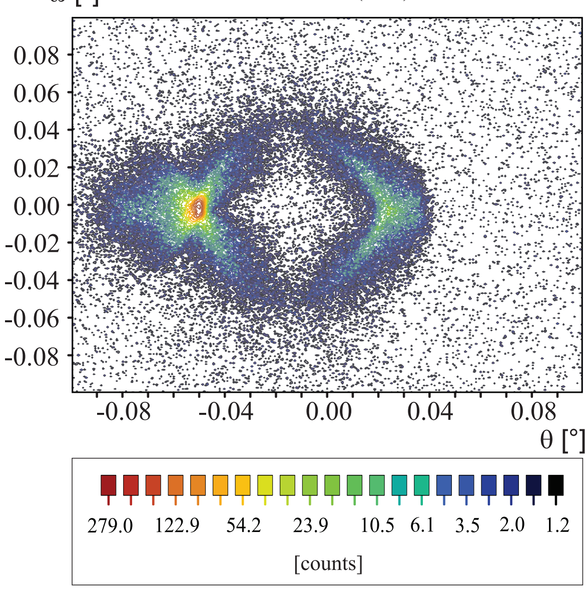

ω/2θ scan on (004) reflection at 5N

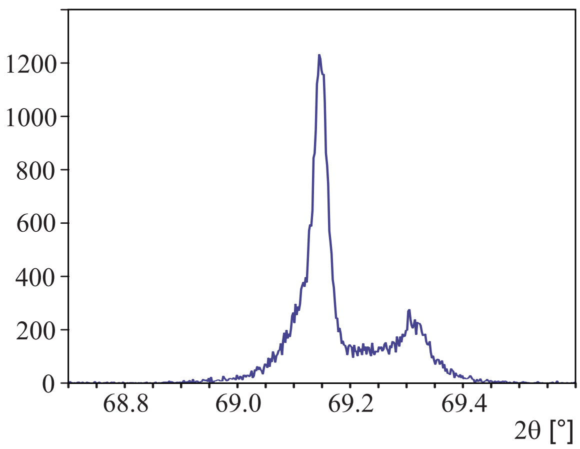

- RSM measurement time: 12h.
- The scan was measured through the center of the RSM at ω=0.

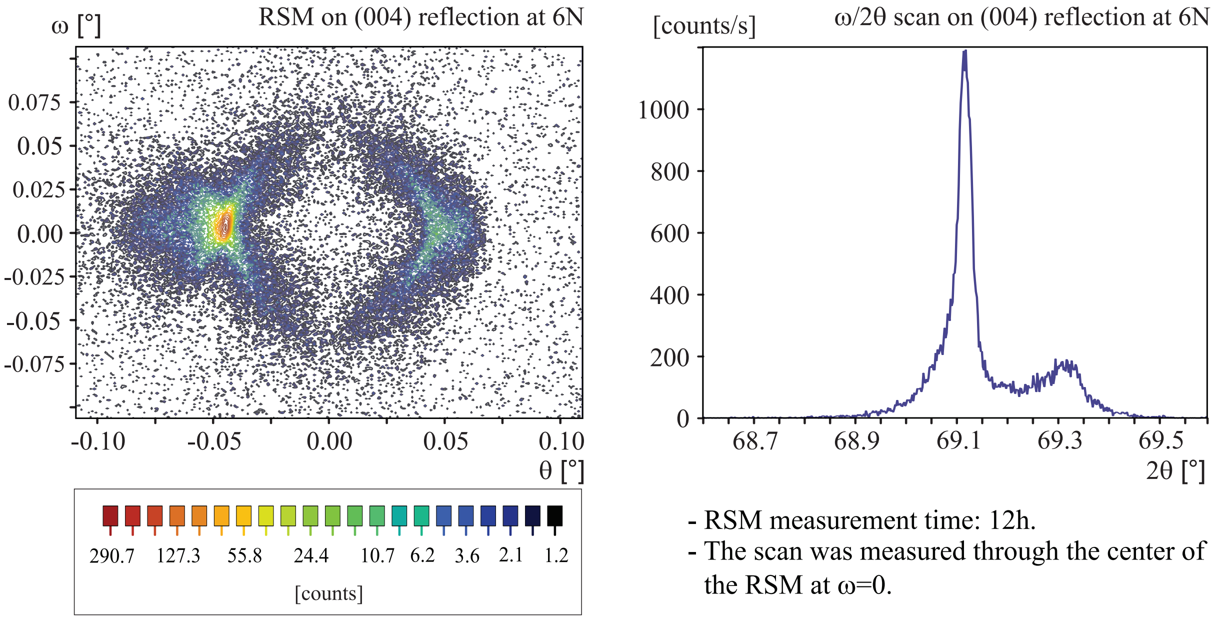

Figure S2 RSM and ω/2θ scans measured on the (004) reflection at the mechanically loaded tensile specimen.

An evaluation of these patterns with respect to loading, peak position and intensity is depicted in Table S1.

Table S1. Evaluation of the RSM-patterns depicted above.

Load [N]	Peak Position	ω [°]	2θ [°]	FWHMx [°]	FWHMy [°]	Intensity [counts]
1.0	left	34.5688	69.1264	0.00326	0.00869	550
	right	34.5832	69.1552	0.00388	0.00908	129
2.0	left	34.5640	69.1264	0.00329	0.00585	701
	right	34.5936	69.1840	0.00453	0.01068	75
3.0	left	34.5680	69.1280	0.00353	0.00938	471
	right	34.6128	69.2144	0.00400	0.01071	53
4.0	left	34.5668	69.1264	0.00310	0.00846	567
	right	34.6260	69.2432	0.00666	0.00894	35
5.0	left	34.5625	69.1256	0.00349	0.00849	310
	right	34.6321	69.2744	0.00312	0.00590	28
6.0	left	34.5638	69.1268	0.00315	0.01015	323
	right	34.6502	69.3092	0.00184	0.00136	21

C. Simulated RSM-patterns on the Si(004) reflection

Related to the introduced approach, the impact of the considered number of element layers on the simulated patterns was investigated by their systematic consideration or omission. Figure S3 schematically depicts the assessed area related to the (004) reflection. The presented figures of are taken from [42].

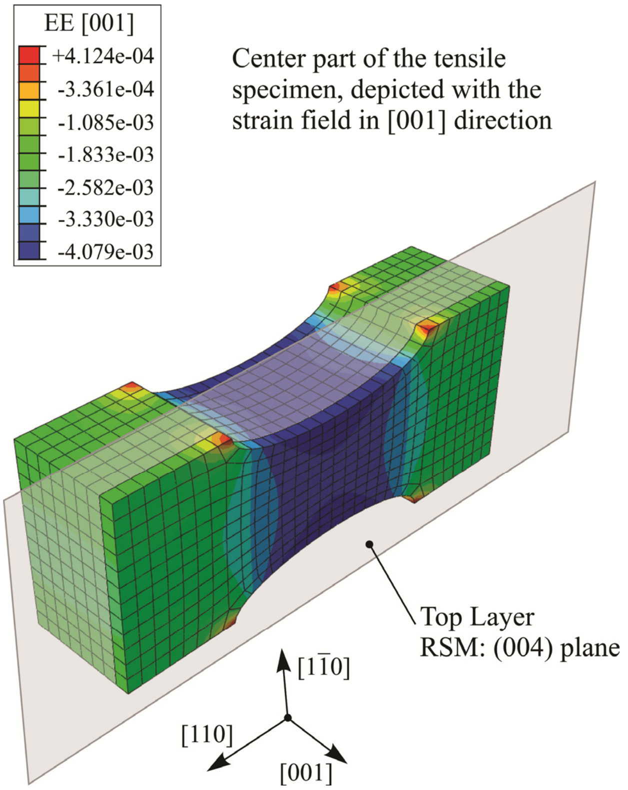

Figure S3 Schematic depiction of the element layers at the specimen central part which were systematically considered for the simulated (004) RSM patterns depicted in Figure S4.

The first two simulated RSM patterns of Fig. S4 are based on all elements of the center part, *cf.* Fig S3. Compared to Fig. 7, a different setting for the estimated energy distribution was used (width of the function), *cf.* Eq. (11) or even omitted, *cf.* Fig. S4 (top left). The consideration of all elements provides several data points outside the applied measurement range for ω and 2θ. Within the (004) RSM measurements, these artifacts are caused by the edge-elements at the transition area between broader and center part. Consequentially the picture sections were defined to be conforming to those of the measured patterns. The same effects were observed within the (440) patterns where the edge-elements were found to be responsible for the remarkable expansions ('arms') in ω-direction, *cf.* Fig S5

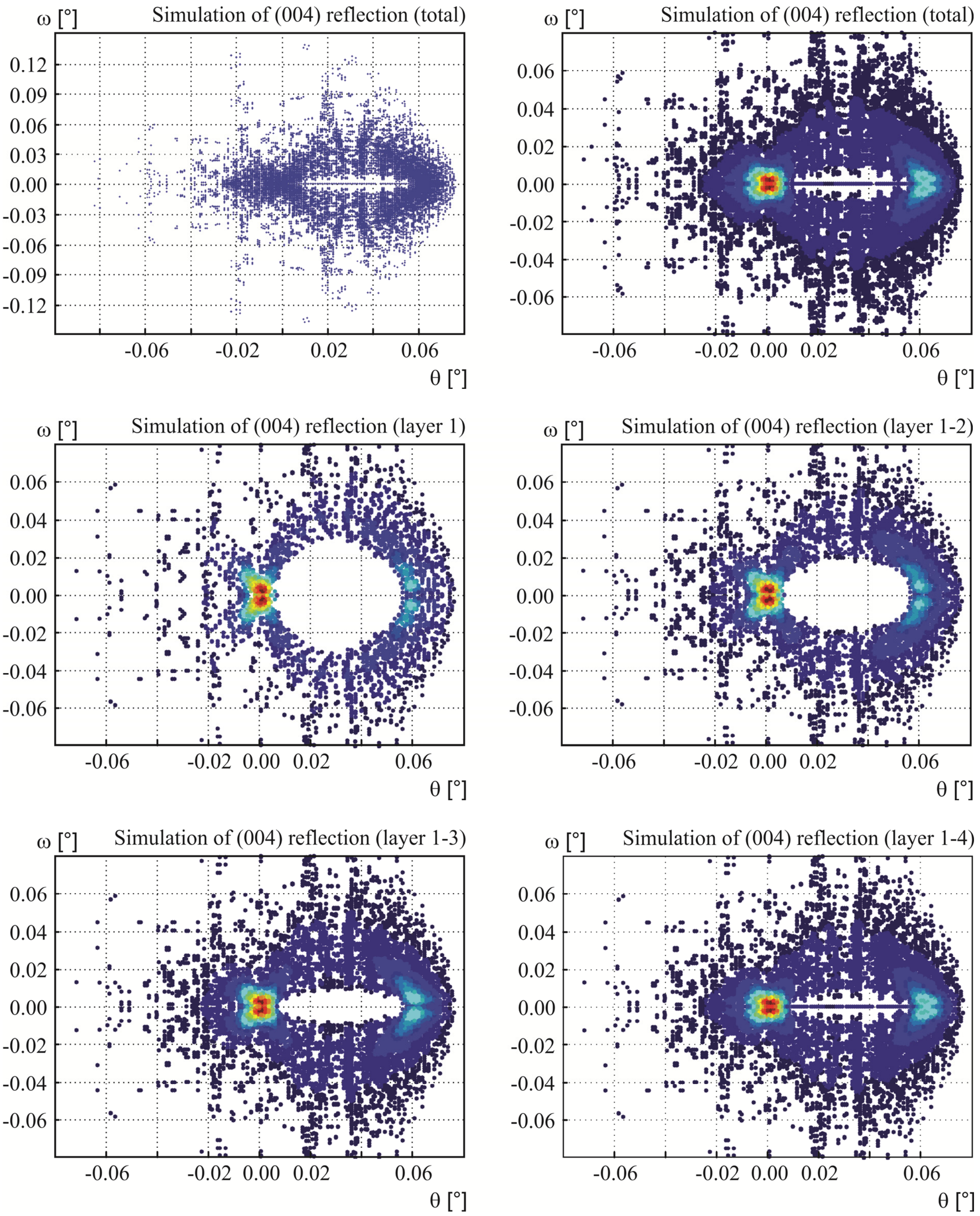

Figure S4 Simulated (004) RSM patterns considering different numbers of FE-elements which have been taken as a basis for the analytical approach.

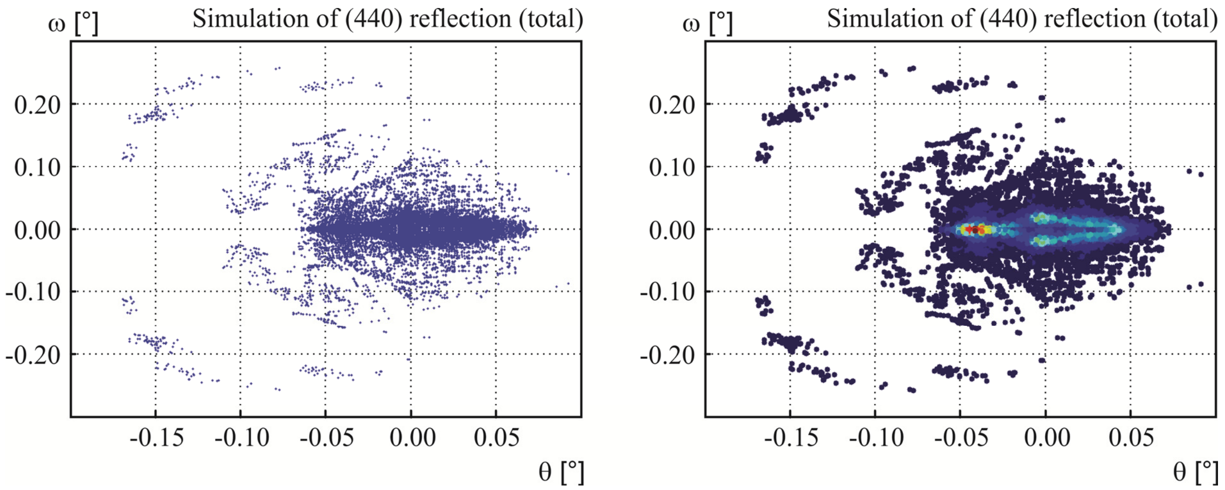

Figure S5 Simulated (440) RSM patterns based on the total number of FE-elements for the analytical approach.

D. Shape Functions

The analytical approach was based on the following *Abaqus* internal definition of elements and shape functions. The isoparametric element coordinates (g, h, r) span a range from -1 to +1 in an element, [21].

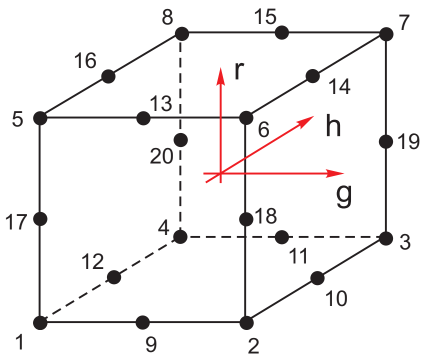

Figure S5. 20-node brick element with the corresponding node numbering.

Shape functions:

$$u_1(g,h,r) = -\frac{1}{8}(1-g)\cdot(1-h)\cdot(1-r)\cdot(2+g+h+r)$$

$$u_2(g,h,r) = -\frac{1}{8}(1+g)\cdot(1-h)\cdot(1-r)\cdot(2-g+h+r)$$

$$u_3(g,h,r) = -\frac{1}{8}(1+g)\cdot(1+h)\cdot(1-r)\cdot(2-g-h+r)$$

$$u_4(g,h,r) = -\frac{1}{8}(1-g)\cdot(1+h)\cdot(1-r)\cdot(2+g-h+r)$$

$$u_5(g,h,r) = -\frac{1}{8}(1-g)\cdot(1-h)\cdot(1+r)\cdot(2+g+h-r)$$

$$u_6(g,h,r) = -\frac{1}{8}(1+g)\cdot(1-h)\cdot(1+r)\cdot(2-g+h-r)$$

$$u_7(g,h,r) = -\frac{1}{8}(1+g)\cdot(1+h)\cdot(1+r)\cdot(2-g-h-r)$$

$$u_8(g,h,r) = -\frac{1}{8}(1-g)\cdot(1+h)\cdot(1+r)\cdot(2+g-h-r)$$

$$u_9(g,h,r) = \frac{1}{4}(1-g)\cdot(1+g)\cdot(1-h)\cdot(1-r)$$

$$u_{10}(g,h,r) = \frac{1}{4}(1-h)\cdot(1+h)\cdot(1+g)\cdot(1-r)$$

$$u_{11}(g,h,r) = \frac{1}{4}(1-g)\cdot(1+g)\cdot(1+h)\cdot(1-r)$$

$$u_{12}(g,h,r) = \frac{1}{4}(1-h)\cdot(1+h)\cdot(1-g)\cdot(1-r)$$

$$u_{13}(g,h,r) = \frac{1}{4}(1-g)\cdot(1+g)\cdot(1-h)\cdot(1+r)$$

$$u_{14}(g,h,r) = \frac{1}{4}(1-h)\cdot(1+h)\cdot(1+g)\cdot(1+r)$$

$$u_{15}(g,h,r) = \frac{1}{4}(1-g)\cdot(1+g)\cdot(1+h)\cdot(1+r)$$

$$u_{16}(g,h,r) = \frac{1}{4}(1-h)\cdot(1+h)\cdot(1-g)\cdot(1+r)$$

$$u_{17}(g,h,r) = \frac{1}{4}(1-r)\cdot(1+r)\cdot(1-g)\cdot(1-h)$$

$$u_{18}(g,h,r) = \frac{1}{4}(1-r)\cdot(1+r)\cdot(1+g)\cdot(1-h)$$

$$u_{19}(g,h,r) = \frac{1}{4}(1-r)\cdot(1+r)\cdot(1+g)\cdot(1+h)$$

$$u_{20}(g,h,r) = \frac{1}{4}(1-r)\cdot(1+r)\cdot(1-g)\cdot(1+h)$$

$$\overline{u}(g,h,r) = \sum_{i=1}^{20} u_i(g,h,r)$$
